# CD16a pairs form the basal molecular subunit for the NK-cell ADCC lytic synapse

**DOI:** 10.1093/jimmun/vkaf077

**Published:** 2025-06-16

**Authors:** Patrick Ross, Tania Cid, Monica Fernández Quintero, Johannes Loeffler, Hijab Fatima, Dan P Leaman, Jessica Matthias, Kathryn Spencer, Michael B Zwick, Scott C Henderson, Andrew B Ward, Emily M Mace, Charles Daniel Murin

**Affiliations:** San Diego Biomedical Research Institute, San Diego, CA, United States; Department of Integrative Structural and Computational Biology, Scripps Research, La Jolla, CA, United States; San Diego Biomedical Research Institute, San Diego, CA, United States; Department of Integrative Structural and Computational Biology, Scripps Research, La Jolla, CA, United States; Department of Integrative Structural and Computational Biology, Scripps Research, La Jolla, CA, United States; Department of Integrative Structural and Computational Biology, Scripps Research, La Jolla, CA, United States; Department of Pediatrics, Columbia University Irving Medical Center, New York, NY, United States; Department of Immunology and Microbiology, Scripps Research, La Jolla, CA, United States; Abberior Instruments America, Bethesda, MD, United States; Core Microscopy Facility, Scripps Research, La Jolla, CA, United States; Department of Immunology and Microbiology, Scripps Research, La Jolla, CA, United States; Department of Integrative Structural and Computational Biology, Scripps Research, La Jolla, CA, United States; Core Microscopy Facility, Scripps Research, La Jolla, CA, United States; Department of Integrative Structural and Computational Biology, Scripps Research, La Jolla, CA, United States; Department of Pediatrics, Columbia University Irving Medical Center, New York, NY, United States; San Diego Biomedical Research Institute, San Diego, CA, United States; Department of Integrative Structural and Computational Biology, Scripps Research, La Jolla, CA, United States

**Keywords:** antibodies, cytotoxicity, Fc receptors, natural killer cells

## Abstract

NK cells utilize effector functions, including antibody-dependent cellular cytotoxicity (ADCC), for the clearance of viral infection and cellular malignancies. While antibody-induced clustering of FcγRIIIa (CD16a) is thought to drive ADCC, the molecular basis for this activity has not been fully described. We used MINFLUX nanoscopy to map the spatial distribution of CD16a within the NK-cell ADCC immune synapse. In both resting and NK cells activated on supported lipid bilayers by Trastuzumab, we detected pairs of CD16a molecules approximately 18 nm apart that could be homodimers. NK-cell activation results in a modest increase of clusters of 4 or more CD16a localizations without a change in cluster characteristics, while CD16a pair distances do not significantly change, suggesting that subtle structural changes underpin ADCC-based activation. Our results provide the highest spatial resolution yet described for CD16a imaging, offering insight into how CD16a organization within the immune synapse could influence ADCC activity.

## Graphical abstract

**Figure:**
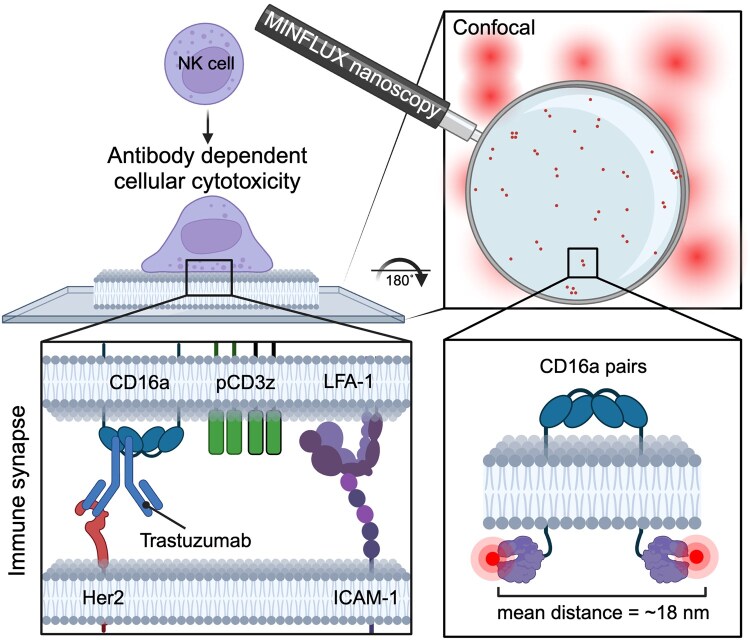
Created in BioRender. Murin, C. (2025) https://BioRender.com/mrto39z.

## Introduction

NK cells are innate immune lymphocytes that play important roles in the clearance of viral infection and cellular malignancies.^1^ A critical effector function of NK cells is ADCC in which CD16a binds to the fragment crystallizable (Fc) domain of IgG within immune complexes on the target cell surface, inducing NK-cell activation and degranulation.^2^ ADCC activity is central to the efficacy of many therapeutic mAbs. Despite the importance of spatial modulation for activating receptor function, a detailed molecular description of CD16a spatial distribution and oligomerization within the cellular context has not been described.

Advancements in light microscopy now allow for the localization of single fluorophores attached to biomolecules with single-digit nanometer precision. MINFLUX localization uses the zero intensity of a donut-shaped excitation beam to gradually approach the fluorophore position.^3^ Here we leverage the precise molecular localization of MINFLUX nanoscopy to describe the spatial distribution of CD16a on the NK-cell membrane. Using a genetically introduced CD16a construct with a SNAP tag on the CT, we observed that several CD16a localizations occurred as 2 localizations within a 40 nm radius (ie pairs). These pairs occur on average of about 18 nm apart and persist on ADCC-activated NK cells. Molecular modeling suggests that these pairs could manifest as homodimers. Our data support a model where pairs of CD16a undergo subtle changes in distribution upon binding to immune complexes within an immune synapse to perform ADCC.

## Materials and methods

### Cell culture

SKOV-3 cells (HTB-77) and NK-92 cells (CRL-2407) were purchased from ATCC (Manassas, Virginia, United States of America). SKOV-3 cells were grown and passaged in DMEM (Gibco) media supplemented with 10% (v/v) FBS (Gibco) and 1X Antibiotic-Antimycotic (Anti-Anti, Thermo Fisher). NK-92 cells were grown and passaged in MyleoCult (STEMCELL Technologies) media supplemented with 10% FBS, 1X Anti-Anti, and 200 IU/mL IL-2 (Peprotech). Cells were maintained for less than 20 passages and regularly tested for mycoplasma.

### Cell line generation

The NK-92^CD16-SNAP^ cell line was constructed using the PiggyBac system.^4^ Briefly, NK-92 cells were co-nucleofected with a CD16-SNAP plasmid and the hyPBase plasmid carrying the transposase (VectorBuilder) at a ratio of 3:1, respectively, using a Lonza P3 Primary Cell kit and a Lonza 4D-nucleofector device with the program CA137. Cells were allowed to recover and then sorted for CD16a expression using a Sony MA900 or Thermo Fisher Scientific Bigfoot cell sorter and expanded.

### Isolation of primary NK cells

PBMCs were first isolated from whole blood using a SepMate PBMC Isolation spin column, according to the instructions (STEMCELL Technologies). NK cells were then isolated using an EasySep Human NK Cell Isolation Kit (STEMCELL Technologies) according to the instructions and used immediately.

### ADCC assay

NK-92^CD16-SNAP^ ADCC activity was measured using an Agilent xCELLigence Real-Time Cell Analysis (RTCA) SP impedance instrument that was set up in a stationary 37 °C incubator with 5% CO_2_. SKOV-3 cells were added to an E-Plate 96 PET plate (Agilent) and after ∼24 h, Traz (Selleckchem) was added followed by NK cells at an E:T of 5:1. Impedance was measured every 15 min for 24 h. ADCC activity was determined using the Agilent xCELLigence immunotherapy software, and curves were plotted using Prism 10 (GraphPad).

### Liposome production

Liposomes were formed by dissolving 1-palmitoyl-2-oleoyl-glycero-3-phosphocholine (POPC) and 1,2-dioleoyl-sn-glycero-3-[(N-(5-amino-1-carboxypentyl)iminodiacetic acid) succinyl] (nickel salt) (DGS-NTA(Ni)) (Avanti) in chloroform at a 96:4 molar ratio. Lipids were dried under vacuum overnight, hydrated, and then sonicated for 30 s. Liposomes were then extruded sequentially through 0.8-μm–0.1-μm filters using a Mini Extruder (Avanti) at RT.

### Immunological synapse formation on glass

Cover glasses (Corning, 18 mm, #1.5) were coated with an anti-β2 integrin (α-CD18) antibody (5 µg/mL in PBS) or α-CD16/α-CD18 antibodies (5 µg/mL in PBS) for 30 min at RT and washed with PBS before adding NK-92^CD16-SNAP^ cells (200 µL at ∼1 × 10^6^ cells/mL) and placed in an incubator at 37 °C and 5% CO_2_ for 30 min. Medium was removed and the sample was washed with PBS. Cells were then fixed, permeabilized, post-fixed, and blocked with Image-iT (Invitrogen) for 30 min. SNAP-tagged CD16a was labeled with 1 µM AF647-SNAP (New England Biolabs) in PBS for 50 min. Samples were then washed and blocked with PBS containing 5% (w/v) BSA for ∼2 h. Samples were then incubated with an α-pCD3ζ antibody (BD Biosciences, clone K25-407.69, AF488, 1:50) at 4 °C overnight. Subsequently, samples were washed, post-fixed, and mounted with ProLong Diamond (Invitrogen).

### Immunological synapse formation on SLBs

SLBs were prepared on cover glasses (Deckgläser coverslips, #1.5) that were thoroughly cleaned. Coverslips were then oxygen plasma (Solarius) treated before being attached to a 6-channel sticky-slide (Ibidi VI 0.4). Liposomes were deposited into each channel, incubated for 20 min, and washed with PBS. SLBs were incubated with 100 µM NiCl_2_ containing 1% (w/v) BSA for 20 min, washed, and then incubated with His-tagged HER2 (10 µg/mL; Acro Biosystems) and His-tagged ICAM-1 (1 µg/mL; Acro Biosystems) for 60 min at 37 °C, and then finally washed with PBS. SLBs were then incubated with either Traz (Selleckchem) at 10 µg/mL or PBS alone and washed. Next, 2 × 10^5^ NK cells were applied and incubated at 37 °C in 5% CO_2_ for 5 min. Samples were then washed, fixed with 4% PFA, and blocked with Image-iT (Thermo Fisher). CD16a was labeled with 2 µM AF647-SNAP for 50 min, washed and the sample subsequently probed with an α-pCD3ζ antibody at 4 °C overnight.

### Confocal image collection

Confocal images of NK cells stained for SNAP-CD16 and pCD3ζ were acquired on a Zeiss LSM 780 laser scanning confocal microscope. A series of images of ∼2 µm in the z-plane were collected with a step size of 200 nm for each cell to cover the volume of the immunological synapse. The z-plane image determined to be in focus (i.e. closest to the imaging plane) was then used for quantification. All confocal image analysis was performed using Imaris 10.1 particle extraction with dynamic background. Data were filtered to remove small particles of a few pixels.

### MINFLUX sample preparation

Samples for MINFLUX data collection were prepared with AF647-labeled NK-92^CD16-SNAP^ as described above for confocal microscopy. Cells were subsequently labeled with phalloidin (Invitrogen, AF488, 1:200) to assist in finding ROIs in confocal mode. Before mounting, 150 nm diameter gold beads (AUROlite Au/TiO_2_, STREM) were added to the samples. Cover glasses were placed onto glass slides featuring a cavity well filled with GLOX buffer (50 mM Tris-HCl, 10 mM NaCl, 10% [w/v] glucose, 10 mM cysteamine, 40 µg/mL bovine-liver catalase, and 100 µg/mL glucose oxidase from *Aspergillus niger*, type VII, pH 8.0) and sealed.

### Image collection: MINFLUX

All MINFLUX data were acquired on a commercial MINFLUX microscope using the Imspector software with MINFLUX drivers (Abberior Instruments). The sample position in relation to the microscope was actively stabilized on the backscattering of a 975-nm-widefield illumination by the gold beads (msd < 3 nm for all 3 axes).^5^ On-state fluorophores within ROIs were driven into the dark state through iterative confocal scanning of the 640-nm laser. This region of interest (ROI) was imaged using the standard 2D MINFLUX imaging sequence with a 640-nm-excitation power of 13 µW. During the targeting sequence, the excitation laser power was ramped up with each iteration. Reconstructions containing trace centers (groups of individual localizations originating from the same emission event) were reconstructed with 10-nm circles.

### Data analysis MINFLUX

MINFLUX data were filtered to remove individual localizations with effective frequencies in offset (efo, background-corrected emission rates) greater than 50,000 Hz and center frequency ratios (cfr, ratio between photon count detected during TCP center exposure and photon count detected during TCP offset exposures) greater than 0.95, and traces with fewer than 3 localizations were removed. The center of each trace was then found using Density-Based Spatial Clustering of Application with Noise (DBSCAN)^6^ with a search radius of 4 nm and a threshold of 3 localizations, yielding 2D coordinates for further analysis.

Cluster analysis was performed using the RSMLM^7^ package to identify clusters with 4 localizations in a search radius of 40 nm. Nearest neighbor analysis was performed using the ball tree algorithm in Scikit-learn.^8^ The nearest neighbor of isolated pairs was determined by identifying clusters of 2 trace centers in a 40-nm search radius using DBSCAN and then filtered using the ball tree algorithm in Scikit-learn.^8^ Ripley H analysis was completed as previously described, implemented as a custom function in Python.^9^

### AlphaFold 3 modeling

We predicted the dimeric structure of CD16a (UniProt ID P08637) using the AlphaFold 3 server.^10^ The AlphaFold 3 structure model was aligned in the membrane using the PPM server^11^ and inserted into a plasma membrane consisting of POPC and cholesterol in a 3:1 ratio, using the CHARMM-GUI Membrane Builder.^12^ We added an additional model exclusively predicting the CT of CD16a (starting from ASP239) fused to the SNAP-tag. Using the resulting model, we performed 100 ns of molecular dynamics simulation in AMBER,^13^ followed by a hierarchical average linkage clustering in cpptraj.^14^ The clustered conformations were reassembled with the membrane-embedded model in MOE^15^ in various orientations and subjected to energy minimization.

### Statistics

Flow cytometry data were compared using an ordinary one-way ANOVA analysis. For all confocal data, SuperPlots were utilized.^16^ Statistical significance of confocal data was calculated using a ratio paired 2-tailed *t* test. Statistical significance of MINFLUX data was calculated using the Mann–Whitney test. All calculations were performed using Prism 10.

## Results and discussion

### NK cells with endogenously tagged CD16a perform ADCC normally

**Figure 1.**
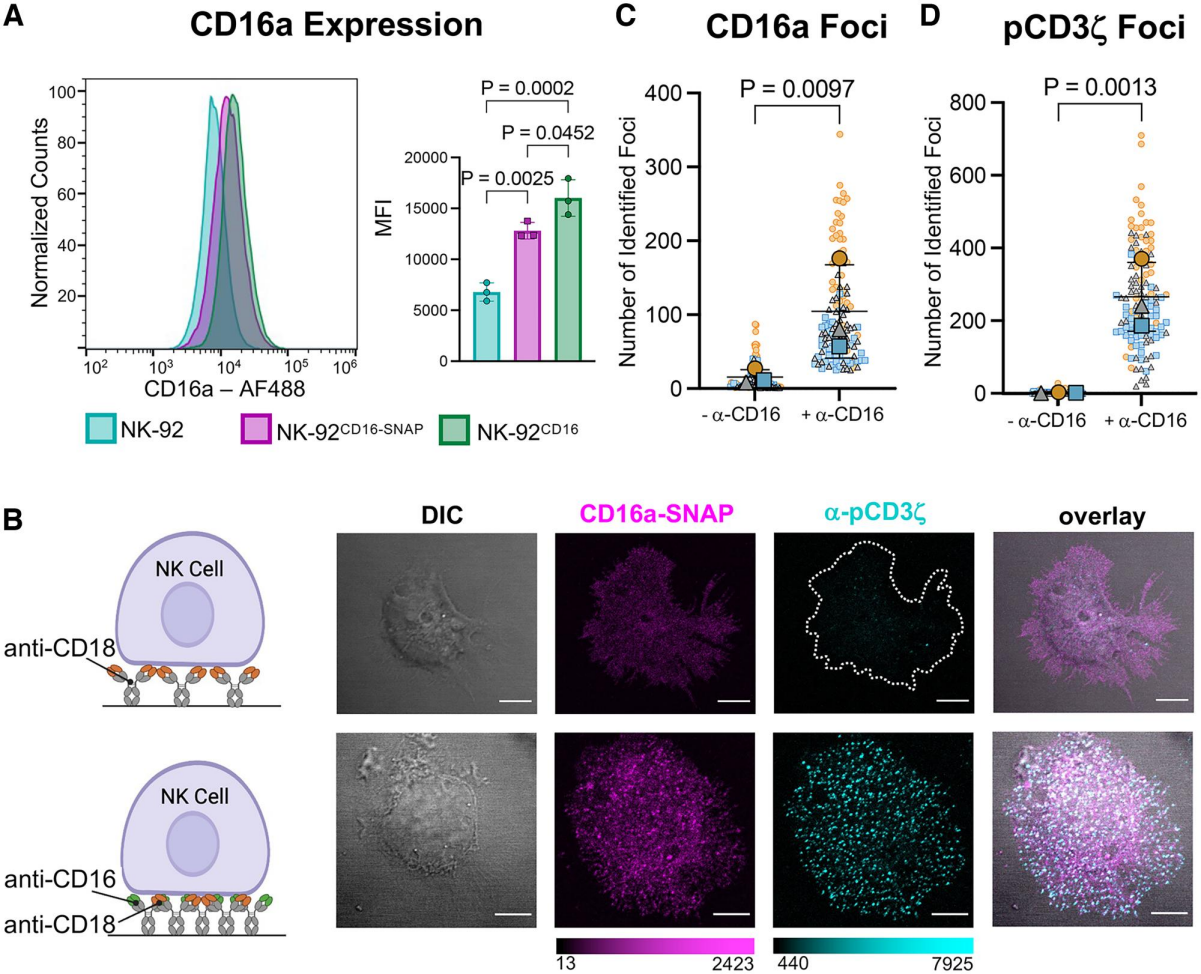
A CT SNAP tag on CD16a does not disrupt ADCC activity. (A) Flow cytometry quantification of CD16a expression on NK-92, NK-92^CD16-SNAP^, and NK-92^CD16^ cells stained with α-CD16 antibody 3G8 (AF488 labeled). (B) The far-left cartoon represents conditions used to tether NK-92^CD16-SNAP^ cells with an α-CD18 antibody (top) and activate via CD16a cross-linking with an α-CD16 antibody (3G8) coated on glass. Then, images from left to right show representative DIC and confocal micrographs of NK-92^CD16-SNAP^ synapses formed on glass coated with α-CD18 antibodies (top panel) or a combination of α-CD16/α-CD18 antibodies (bottom panel) and stained for CD16a-SNAP (AF647) and pCD3ζ (AF488). (C, D) Quantification of the (C) number of CD16a foci and (D) pCD3ζ foci per NK-92^CD16-SNAP^ synapse (without and with CD16a cross-linking). Large symbols in the SuperPlots show the means of 3 independent experiments, with small symbols showing each technical replicate from at least 30 cells per experiment. Scale bars represent 5 µm. Part of this figure was created in BioRender. Murin, C. (2025) https://BioRender.com/45o4od7.

To investigate the organization of CD16a in the NK-cell membrane during the process of ADCC, we generated an NK-92 cell line with a genetically integrated *FCGR3A* gene containing a SNAP-tag on the CT and a control cell line with an untagged version of CD16a via nucleofection with a piggyBac transposase,^17^ which we termed NK-92^CD16-SNAP^ and NK-92^CD16^, respectively. We first confirmed CD16a expression using flow cytometry (CD16a clone 3G8, AF488) (Fig. 1A). We then validated the ADCC activity of our NK-92 cell lines using an impedence-based assay, with SKOV-3 cells as targets, which express HER2 and can be targeted for ADCC via Traz. We observed comparable cytolytic activity between NK-92 cells expressing CD16a alone (Fig. S1A) or the CD16a-SNAP construct (Fig. S1B), and both cell lines displayed comparable activity to NK cells isolated from frozen human PBMCs (Fig. S1C).

To establish the utility of the NK-92^CD16-SNAP^ cell line for downstream microscopy studies, we modeled the NK-cell immune synapse using antibody-coated glass to achieve receptor cross-linking.^18^^,^^19^ We compared synapses formed on glass coated with α-CD16/α-CD18 antibodies or with an α-CD18 antibody alone (Fig. 1B). NK-92^CD16-SNAP^ cells were fixed and probed with a SNAP ligand, which labels SNAP-tagged CD16a, and an antibody targeting phosphorylated CD3ζ (pCD3ζ) as a marker of downstream activation^20^ (Fig. 1B).

The NK-92^CD16-SNAP^ cells ligated on glass dual coated with α-CD16/α-CD18 antibodies produced clearly discernable foci of pCD3ζ where the artificial synapse had been formed (Fig. 1B). The number of CD16a foci in NK cells was higher on glass coated with α-CD16/α-CD18 antibodies compared to the α-CD18 antibody solo-coating (Fig. 1C), and we also observed a greater number of pCD3ζ foci in NK cells activated on glass with α-CD16/α-CD18 antibodies than with α-CD18 alone (Fig. 1D).

### NK cells on supported lipid bilayers with mAb induce increased phosphorylation of CD3ζ

**Figure 2.**
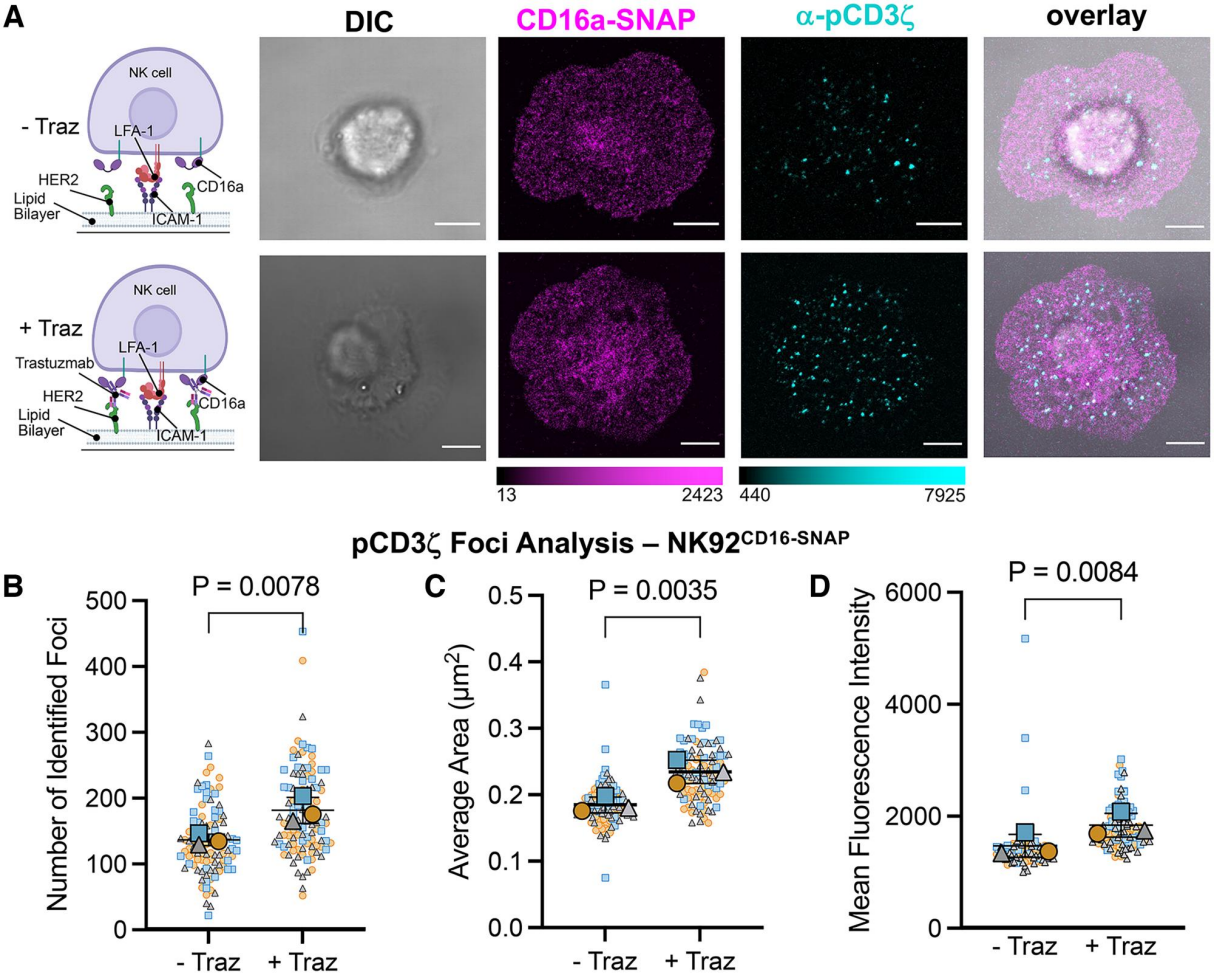
SLBs displaying HER2 and ICAM-1 opsonized with Trastuzumab induce phosphorylation of CD3ζ in NK-92^CD16-SNAP^ cells. (A) The far-left column is a cartoon representation of conditions used to model CD16a-mediated ADCC activation on SLBs containing HER2 and ICAM-1 (top) without Traz and (bottom) with Traz. Then, images from left to right show representative DIC and confocal micrographs of NK-92^CD16-SNAP^ synapses formed on SLBs without Traz (top panel) or with Traz (bottom panel) and stained for CD16a-SNAP (AF647) and pCD3ζ (AF488). (B–D) Quantification of the (B) number, (C) area, and (D) fluorescence intensity of pCD3ζ foci per NK-92^CD16-SNAP^ synapse. Large symbols in the SuperPlots show the means of 3 independent experiments, with small symbols showing each technical replicate from at least 30 cells per experiment. Scale bars represent 5 µm. Part of this figure was created in BioRender. Murin, C. (2025) https://BioRender.com/up8hj62.

To design a model that would recapitulate the immune synapse during ADCC with greater biological relevance, we utilized supported lipid bilayers (SLBs) displaying both HER2 and ICAM-1. After addition of His-tagged HER2 and ICAM-1, bilayer fluidity was confirmed by fluorescence recovery after photobleaching (FRAP) (Fig. S2). We then incubated NK-92^CD16-SNAP^ cells on bilayers and compared them to NK-92^CD16-SNAP^ cells on bilayers that had been opsonized with Traz (Fig. 2A). We observed that the addition of Traz resulted in an increase in the number, size, and intensity of pCD3ζ foci as observed by confocal microscopy (Fig. 2B–D). This suggests that the SLB induced NK-cell activation through CD16a specifically. Visually, we observed no difference in CD16a distribution between the 2 experimental conditions at the resolution of confocal microscopy. We also isolated primary NK cells from healthy human donors and performed identical ADCC activation experiments on SLBs. We observed the same increase in pCD3ζ foci number, size, and intensity upon activation with Traz as we did with our NK-92 cells (Fig. S3), suggesting that our NK-92^CD16-SNAP^ cells function as an appropriate model to study the mechanism of ADCC.

### Pairs of CD16a molecules on resting NK cells redistribute within ADCC synapses formed on an SLB

**Figure 3.**
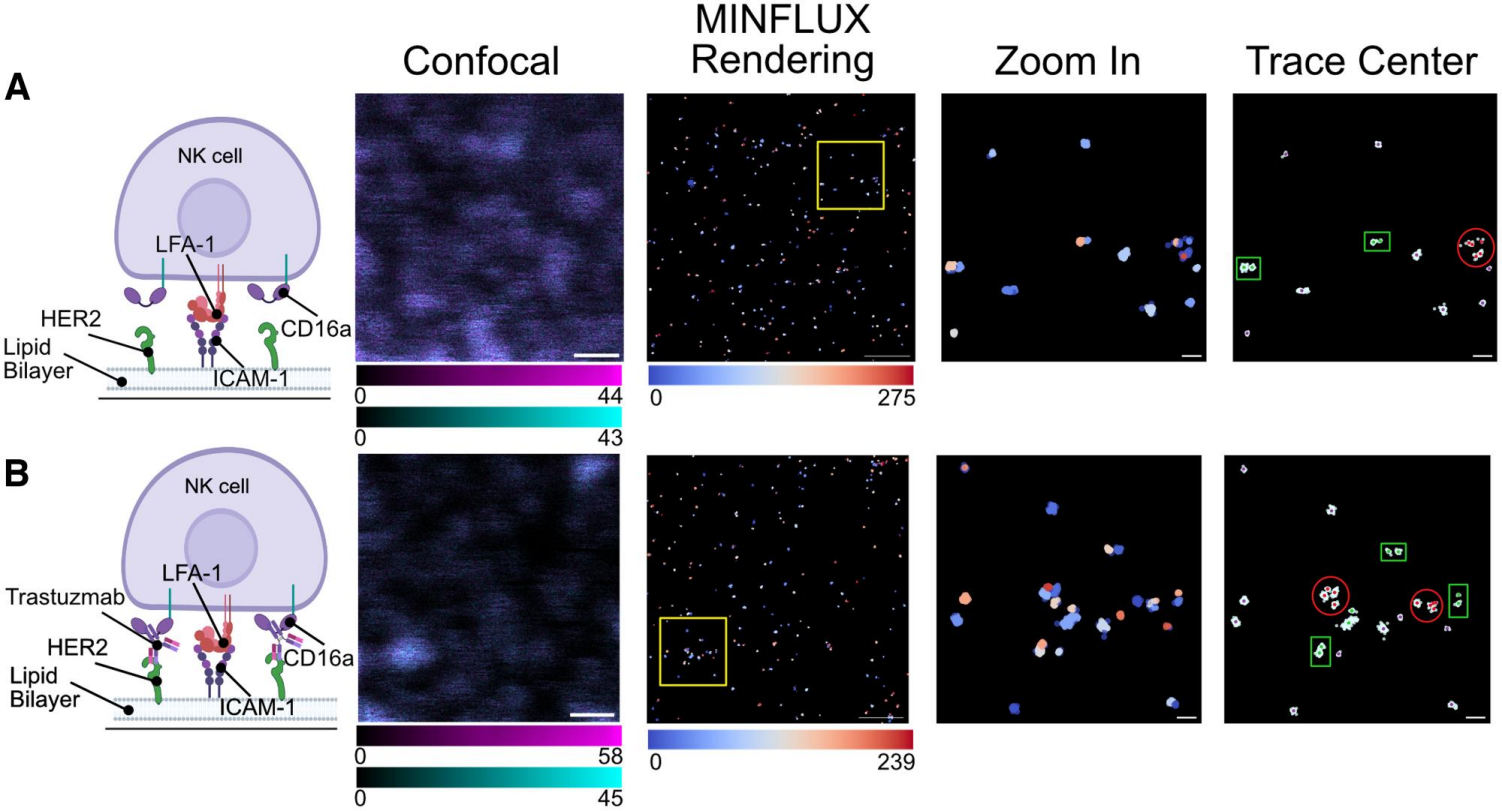
CD16a exists in pairs and small clusters within immune synapses that do not change characteristics upon ADCC activation. (A) The far-left cartoon represents conditions used to model CD16a-mediated ADCC activation on SLBs containing HER2 and ICAM-1 (top) without Traz and (bottom) with Traz. Shown are representative images of NK-92^CD16-SNAP^ confocal and MINFLUX data of immune synapses on SLBs. Data are colored by ID given through DBSCAN. Trace centers are overlaid on raw MINFLUX data (colored in light cyan), and individual traces are colored in purple, isolated pairs in green, and clusters of 4 or more localizations in red. Scale bars in confocal images and raw MINFLUX data represent 500 nm, and zoomed-in-region scale bars represent 50 nm. MINFLUX data were collected across at least 3 independent experiments; n = 12 cells total per condition. Part of this figure was created in BioRender. Murin, C. (2025) https://BioRender.com/72whps6.

To define how CD16a organizes and distributes across the NK-cell membrane, we turned to MINFLUX nanoscopy. We first created artificial immune synapses between NK-92^CD16-SNAP^ cells and SLBs, both in the absence and presence of Traz, fixed and probed cells with SNAP-AF647, and subsequently performed MINFLUX data acquisition (Fig. 3A). Our MINFLUX analysis pipeline mapped individual fluorophores as groups of several localizations, and throughout our data collection, we regularly obtained a mean localization precision of ∼2.5 nm. To determine if CD16a forms clusters, we used Ripley H in which a value >0 represents increased clustering and a value <0 represents dispersion.^9^ We observed that CD16a is not randomly dispersed but organized, and we observed little difference in the degree of clustering between the 2 conditions based on Ripley H function (Fig. S4A).

MINFLUX imaging consistently identified pairs of localized fluorophores across all SLB data sets, which we initially identified visually (Fig. 3). We found little difference in the average nearest neighbor distance between the 2 conditions (Fig. S4B). When we isolated these pairs and performed nearest neighbor analysis of the 2D MINFLUX data, we observed an intra-fluorophore distance of 17.8 nm and 18.8 nm on NK cells activated without and with Traz, respectively (Fig. S4C). These measurements, however, do not account for the possibility of variances in intra-fluorophore distances along the *z* axis, which are projected onto the imaging plane and thus not readily detected in 2D analysis. We used 2D over 3D MINFLUX acquisition because our synapses are generally flat and a 2D acquisition ran more efficiently with fewer rejections of localization attempts due to fluorophore quenching. To our knowledge, this is the most precise localization of CD16a within the NK cell yet shown and the first time we have direct knowledge of the oligomeric state of individual CD16a molecules within the cellular context.

To further describe the clusters of localized fluorophores, we performed Density-Based Spatial Clustering of Application with Noise (DBSCAN)^6^ analysis to identify clusters with 4 or more localizations in a 40-nm search radius. CD16a localizations showed an increase in the number of such clusters in the presence of Traz (Fig. S4D). However, the characteristics of these clusters, including composition, size, and density, were similar between NK-cell immune synapses without and with Traz (Fig. S4E–G). It is not possible to discern which CD16a molecules are bound to immune complexes, and which are unbound using our current experimental setup. However, by labeling CD16a via a CT SNAP tag, we do not interrupt binding to IgG. Therefore, it is possible that the increased clustering is associated with immune complex binding to CD16a pairs (Fig. S4D). Previous work suggests that activation of NK cells by CD16a is through the formation of CD16a clusters on the cell membrane.^18^^,^^21^ However, the small, clustered regions we observe here do not resemble the large, dense clusters observed for other NK-cell receptors (Fig. 3A).^22^^,^^23^

We should note that in these studies, inherent labeling error provides a degree of uncertainty in accurately measuring the distance between receptors. Additionally, an effective labeling efficiency inherently below 100% limits a complete quantitative description of CD16a oligomeric states and spatial distribution.^24^^,^^25^ Indeed, several CD16a localizations were detected in isolation (Fig. 3A).

### Modeling of CD16a pairs suggests possible organizational motifs of the NK-cell ADCC architecture

**Figure 4.**
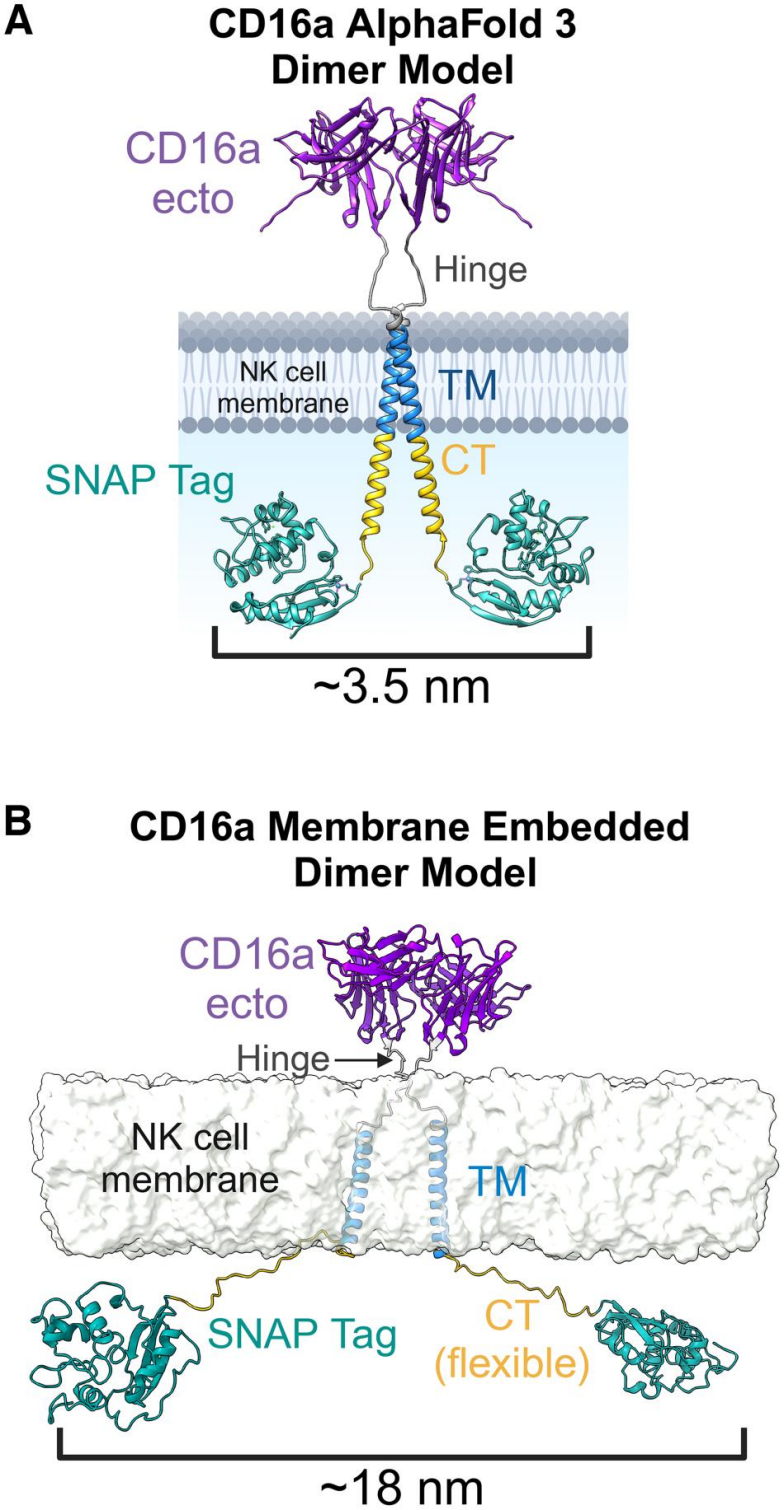
Modeling of CD16a dimers is consistent with MINFLUX localization and shows possible arrangements of the NK-cell ADCC activation complex. (A) Liganded SNAP tags (PDB 3KZZ) were modeled onto the AlphaFold 3 CD16a dimer model at the CT (where they were engineered in our NK-92^SNAP-CD16^ cell line) and the distance measured from their fluorophores. (B) The AlphaFold 3 model was generated with a SNAP tag, a flexible CT, and embedded in a simulated cell membrane before performing global relaxation. We then measured the distance between the relative position of the SNAP-liganded fluorophore. Part of this figure was created in BioRender. Murin, C. (2025) https://BioRender.com/82w6bsu.

We next aimed to reconcile our empirically derived data of CD16a-pair distance measurements within the cellular environment with molecular modeling to determine potential organizational motifs of the ADCC activation complex. We first determined structures of full-length CD16a using AlphaFold 3 containing 2 copies.^10^ The 5 models predicted by AlphaFold 3 all converged on nearly identical structures. Using UCSF Chimera,^26^ we next modeled SNAP tags on the CT of the predicted CD16a dimer at maximal distance apart and determined the potential intra-fluorophore distance to be ∼3.5 nm (Fig. 4A). This falls within the lower range of intra-fluorophore pair distances observed in the MINFLUX data sets (Fig. S4C).

The CT of the CD16a AlphaFold 3 models were modeled as alpha helices, but there are phosphorylation sites within the CT tails that would be more accessible in a disordered motif.^27^ We therefore added nonhelical CT domains (cluster representatives from molecular dynamics simulations) connected to the SNAP tags onto the existing AlphaFold 3 model’s TM regions and relaxed the combined models embedded in a membrane. From the simulations, the CT domains linked to the SNAP tags could adopt a diverse range of distances, including distances around ∼18 nm (Fig. 4B), in-line with the distances observed in the MINFLUX data (Fig. 3D). Thus, the range of distances observed via MINFLUX could be influenced by the flexibility of the CT domains. But the SNAP tags are never close enough to interact directly and thus are unlikely to be responsible for the pairs we observe.

In our AlphaFold 3 model, the CD16a ectodomains form non-covalent, antiparallel homodimers. However, predictions of the CD16a ectodomain homodimers with other available machine-learning–based algorithms such as Chai-1^28^ and Boltz-1^29^ revealed different potential dimeric interfaces. While the ectodomains can probably form dimers, we did not find any clear structural or evolutionary couplings to further confirm the predictions of the CD16a ectodomain homodimers.^30^ It is possible that a homodimer is formed via the TM domains or with CD3ζ, a homodimer that is required for cell surface trafficking of CD16a.^31^^,^^32^ The significant increases in CD3ζ phosphorylation we observed could be driven by changes in its interaction with CD16a within the immune synapse (Fig. 2). CD2 has also been shown to non-covalently interact with CD16a.^33^^,^^34^

The models discussed above are still largely speculative, including the structural nature of the CD16a pairing we observed, and will require additional studies using the tools developed here, including multichannel MINFLUX nanoscopy and structural biology techniques like cryo-EM, to elucidate more precisely the protein orchestration and structure of CD16a in resting versus activated NK cells. These studies will also reveal how additional signaling proteins interplay within the immune synapse to direct ADCC-specific activation as opposed to other forms of NK-cell activation. Our observation of CD16a pairs sheds new light on the molecular orchestration of CD16a on NK cells. Overall, our current findings support a model where CD16a forms molecular clusters upon activation through slight changes in its distribution on the cell membrane, potentially influenced by other molecular interactions including other cell surface receptors or signaling partners.

## Supplementary Material

vkaf077_Supplementary_Data

## Data Availability

The data underlying this article will be shared on reasonable request to the corresponding author.
