# Supplementary material for: CD16a pairs form the basal molecular subunit for the NK-cell ADCC lytic synapse
Source: J Immunol. 2025 Jun 16;214(9):2180–8. doi: 10.1093/jimmun/vkaf077 (PMC12481035; doi:10.1093/jimmun/vkaf077)
Supplement: vkaf077_Supplementary_Data [file vkaf077_supplementary_data.pdf]

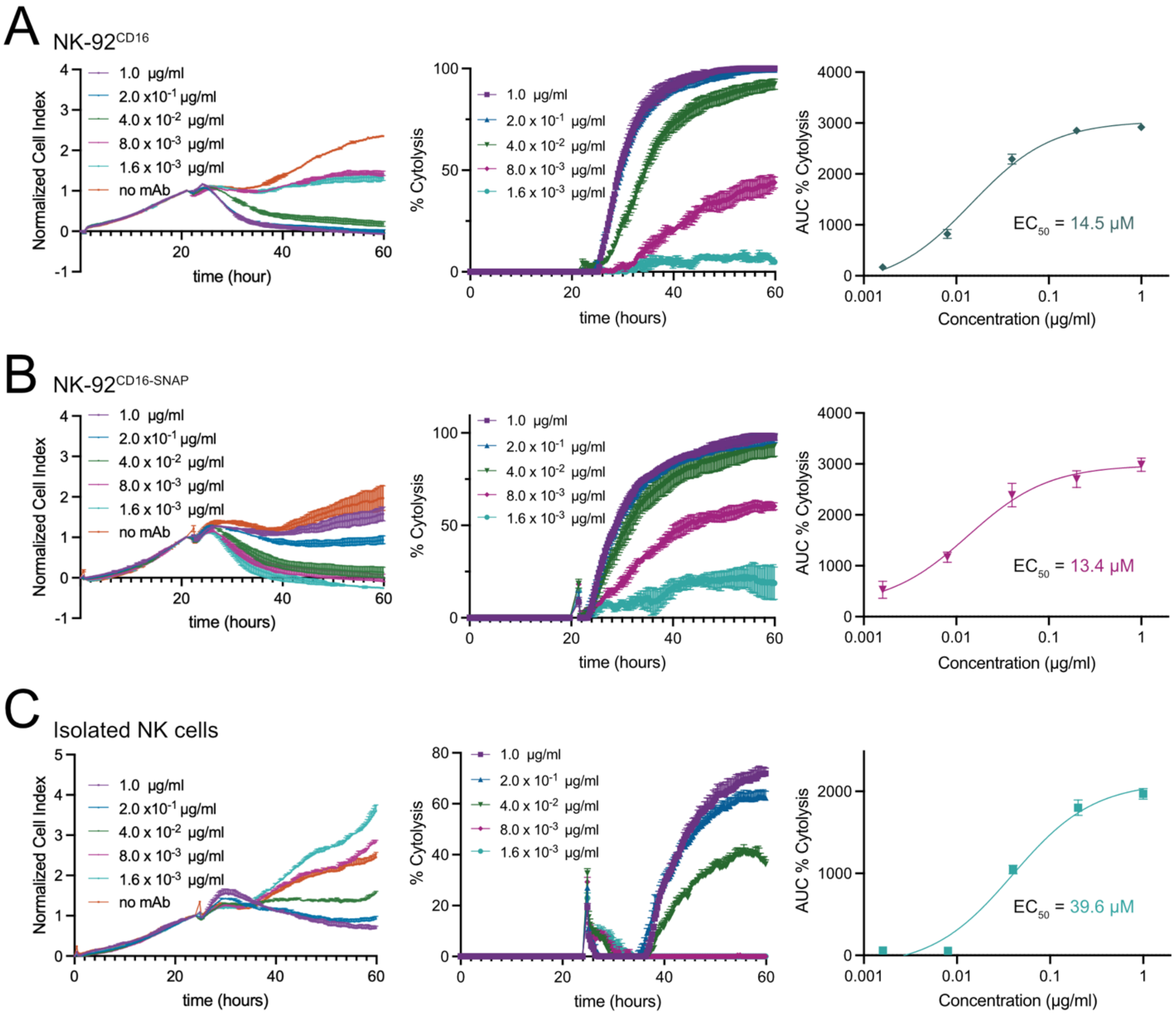

Supplemental Figure 1: Comparison of NK-92^{CD16}, NK-92$^{CD16\text{-}SNAP}$, and isolated NK cell ADCC activity. Representative data from impedance based ADCC activity. From left to right, raw normalized cell index, % cytolysis, and area under the curve (AUC) of % cytolysis for (A) NK-92^{CD16}, (B) NK-92$^{CD16\text{-}SNAP}$, and (C) isolated NK cells from PBMCs.

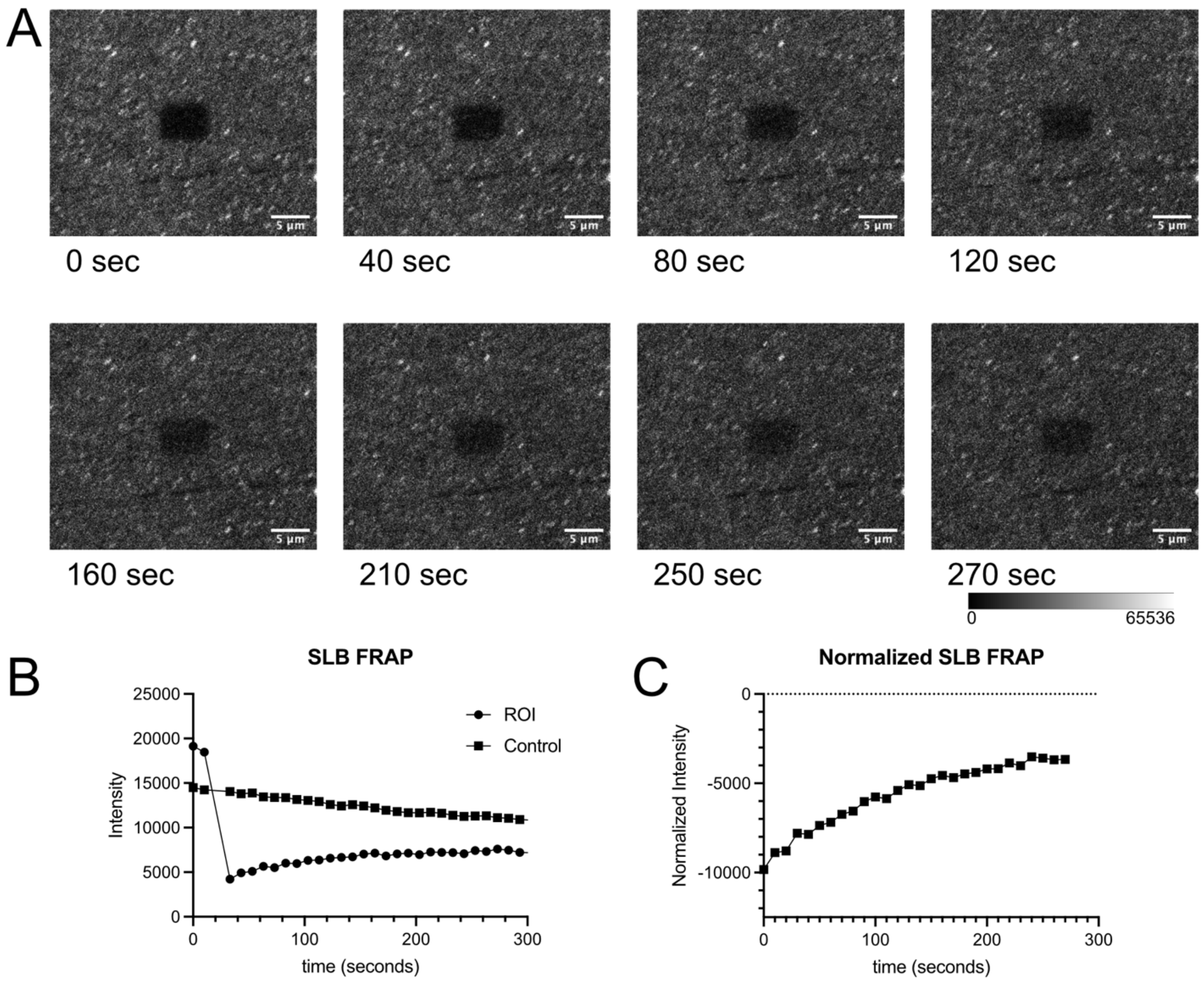

Supplemental Figure 2: Supported lipid bilayers present mobile antigens. SLBs were first prepared as described above. Subsequently, an α-Her2 antibody (R&D systems research grade trastuzumab biosimilar, AF488, 10µg/mL, 1:100) was added and incubated for 30 minutes at RT and washed. Labeled SLBs were imaged on a Zeiss LSM 880 laser scanning confocal microscope, using Perfect Focus. After two scans, a region of interest (ROI) of ~25 μm^2 was photobleached and subsequent fluorescence recovery was recorded. (A) Representative image series acquired during FRAP experiment. (B) Raw fluorescence intensity from FRAP data. (C) Normalized fluorescence intensity presented in B.

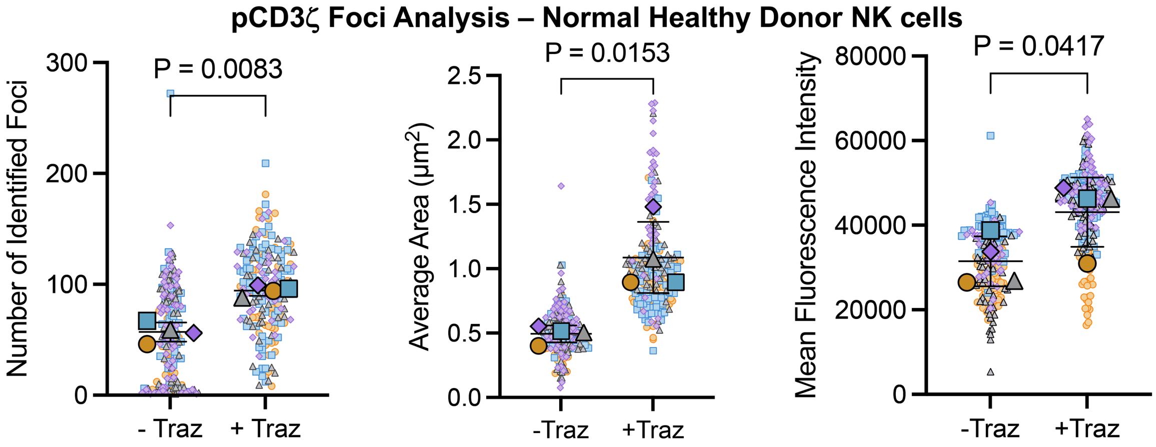

Supplemental Figure 3: SLBs displaying HER2 and ICAM-1 opsonized with Trastuzumab induce phosphorylation of CD3ζ in healthy donor NK cells. NK cells from four healthy donors were used to generate immune synapses on SLBs containing HER2 and ICAM-1, without (-Traz) and with Trastuzumab (+ Traz). Cells were fixed and stained for pCD3ζ (AF488). Shown is the quantification of the number, area and fluorescence intensity of pCD3ζ foci per NK cell synapse. Large symbols in the SuperPlots show the means of three independent experiments with small symbols showing each technical replicate from at least 30 cells per experiment. Each donor is represented by a different symbol.

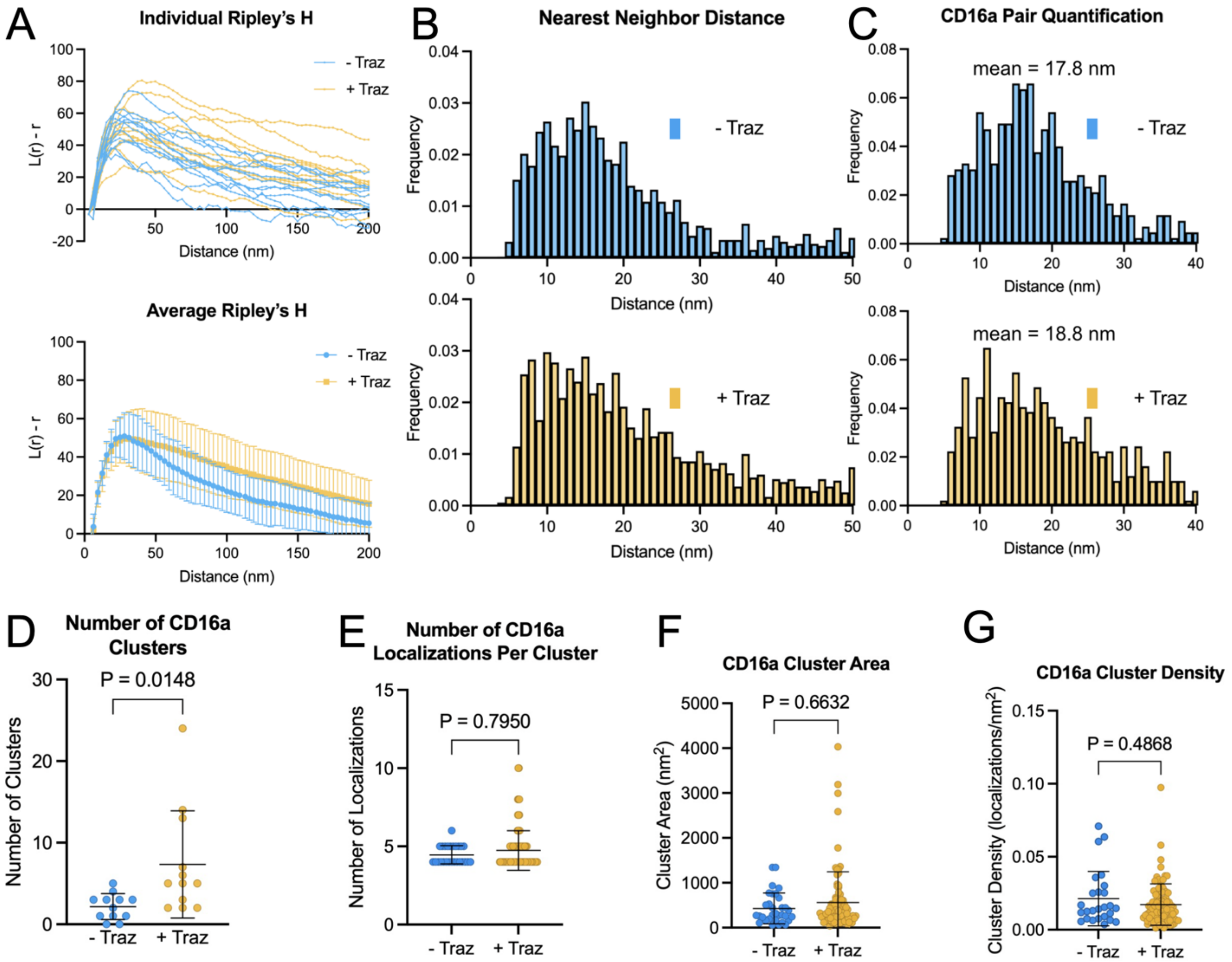

Supplemental Figure 4: Quantification of MINFLUX data from Figure 3. (A) Individual region of interest (ROI) Ripley's H function (top) and average Ripley's H function (bottom) of all MINFLUX data. **(B)** Nearest neighbor analysis of all MINFLUX data. **(C)** Nearest neighbor analysis of isolated pairs of CD16a molecules (2 localizations within a 40 nm radius). **(D)** Quantification of the number of CD16a clusters (4 or more localizations within a 40 nm radius) in each cell. **(E)** Quantification of the number of CD16a localizations in each cluster. **(F)** Measurement of the area of each CD16a cluster. **(G)** Quantification of the density of CD16a localizations per cluster.
